# Deep learning for predicting the occurrence of tipping points

**DOI:** 10.1098/rsos.242240

**Published:** 2025-07-16

**Authors:** Chengzuo Zhuge, Jiawei Li, Wei Chen

**Affiliations:** ^1^ School of Mathematical Sciences, Beihang University, Beijing 100191, People's Republic of China; ^2^ Key Laboratory of Mathematics, Informatics and Behavioral Semantics (LMIB), Beihang University, Beijing 100191, People's Republic of China; ^3^ School of Artificial Intelligence, Beihang University, Beijing 100191, People's Republic of China; ^4^ Zhongguancun Laboratory, Beijing 100194, People's Republic of China; ^5^ School of Systems Science, Beijing Normal University, Beijing 100875, People's Republic of China

**Keywords:** complex systems, machine learning, bifurcation theory, tipping points, nonlinear dynamics

## Abstract

Tipping points occur in many real-world systems, at which the system shifts suddenly from one state to another. The ability to predict the occurrence of tipping points from time series data remains an outstanding challenge and a major interest in a broad range of research fields. Particularly, the widely used methods based on bifurcation theory are neither reliable in prediction accuracy nor applicable for irregularly sampled time series which are commonly observed from real-world systems. Here, we address this challenge by developing a deep learning algorithm for predicting the occurrence of tipping points in untrained systems, by exploiting information about normal forms. Our algorithm not only outperforms traditional methods for regularly sampled model time series but also achieves accurate predictions for irregularly sampled model time series and empirical time series. Our ability to predict tipping points for complex systems paves the way for mitigation risks, prevention of catastrophic failures and restoration of degraded systems, with broad applications in social science, engineering and biology.

## 1. Introduction

Many real-world systems, ranging from biological to climate and financial systems, can experience sudden shifts between states at critical thresholds which are so-called tipping points, for example, the epileptic seizures [1], abrupt shifts in ocean circulation [2,3] and systemic market crashes [4]. Accurately predicting tipping points before they occur has many important applications, including developing strategies to prevent disease outbreak, avoiding disasters induced by climate change and designing robust financial systems. However, due to the complexity of various real-world systems, it is a challenging task to invent an effective tool to predict the occurrence of tipping points [5,6].

A classical mathematical tool to understand tipping points is bifurcation theory which focuses on how dynamical systems undergo sudden qualitative changes as a parameter crosses a threshold [7]. There are basically two classes of methods based on bifurcation theory to deal with tipping points prediction. The first class of methods is using lag-1 autocorrelation which is based on ‘critical slowing down’, a paramount clue as to whether a tipping point is approached [5,8]. Such methods are widely used in various complex systems, including degenerate fingerprinting [9], BB method [10] and ROSA [11]. The second class of methods is using dynamical eigenvalue (DEV) [12], which is based on Takens embedding theorem [13]. Yet the success of the existing methods based on bifurcation theory is impaired by two fundamental limitations. First, these methods are not applicable for irregularly sampled time series data which are commonly observed from various scientific fields, such as geoscientific measurements [14,15], medical observations [16] and biological systems [17]. Second, the performance of existing methods based on bifurcation theory in prediction accuracy is affected by several approximations used in these methods. One such approximation is that only the first-order term of the dynamical systems is considered in these methods, while the impact of the higher-order terms is ignored. However, the higher-order terms become significant for prediction when a tipping point is approached [18] (electronic supplementary material, note S1). Besides, in the approximation of fast-slow systems [19], the delay on the bifurcation-tipping due to the changing rate of the bifurcation parameter is ignored [20,21] (electronic supplementary material, note S2).

Recent studies show that some machine learning-based techniques have been used as effective early warning signals (EWS) of tipping points by learning generic features of bifurcation [18,22–24]. However, they cannot be used for predicting where the tipping points occur. A machine learning framework based on reservoir computing has been developed for tipping points prediction [25–27]. This algorithm requires time series sampled from all interacting variables of study systems for training and prediction. However, this information is often not available for real-world systems. Therefore, it is often not feasible to use this machine learning framework for predicting tipping points of real-world systems.

**Figure 1.**
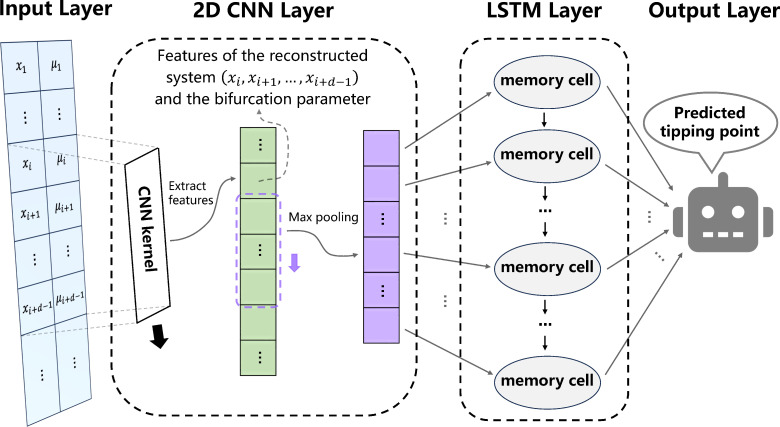
The two-dimensional CNN-LSTM architecture. The two-dimensional convolution kernel transforms the state series and the bifurcation parameter series into a one-dimensional feature series, representing the reconstructed system $\left(x_{i},x_{i+1},\ldots ,x_{i+d-1}\right)$ along with the bifurcation parameter. This features series is then subjected to local max pooling, followed by analysis using an LSTM layer. Finally, the features processed by the LSTM are mapped to the predicted tipping point.

To circumvent the above limitations of existing methods for tipping points prediction, we develop a deep learning (DL) algorithm based on two-dimensional Convolutional Neural Network-Long Short Term Memory (CNN-LSTM) architecture. Based on the embedding theorem for irregular sampling [28] (electronic supplementary material, note S3), this DL algorithm only requires the time series of the state and that of the bifurcation parameter from a single variable of a system (figure 1). The convolution kernels in the two-dimensional CNN layer are used to scan the state time series and the parameter time series. According to the embedding theorem, the state space of an m-dimensional dynamical system can be reconstructed from a sequence of time-delayed observations of a single variable if the embedding dimension d>2m. Here, we use convolution kernels with length d to extract the features of a system reconstructed by a d-dimensional delay embedding, which effectively extract the dynamical features of the m-dimensional dynamical system. These convolution kernels move along the time series, extracting features from each segment of the state time series and the bifurcation parameter time series, thereby generating sequences of feature representations. Then the LSTM layer is used to identify long-term dependencies from the sequences of features of the reconstructed system and the features of bifurcation parameter for tipping points prediction. We assume that the DL algorithm can detect features that emerge in time series prior to a tipping point, such as the features of the recovery rate in normal forms, which are the temporal patterns extracted from the data associated with the occurrence of tipping points. We apply the DL algorithm to predict the occurrence of tipping points in systems it was not trained on. Our DL algorithm is applicable to both regularly sampled and irregularly sampled time series. We first test this DL algorithm on regularly sampled time series generated by the models from ecology [29–31] and climatology [32–35]. The DL algorithm outperforms traditional methods in prediction accuracy for regularly sampled model time series. We further validate our DL algorithm on irregularly sampled time series generated by the same models and two other models from neuroscience with hysteresis phenomena [36,37]. Finally, we validate our DL algorithm in irregularly sampled empirical data from microbiology [38], thermoacoustics [20] and climatology [39]. In this work, we show that our DL algorithm is effective in dealing with dynamical systems exhibiting fold, Hopf and transcritical bifurcation. We anticipate that our DL algorithm may also be applicable to dynamical systems exhibiting other bifurcation types.

## 2. Methods

### 2.1. Bifurcation theory

**Figure 2.**
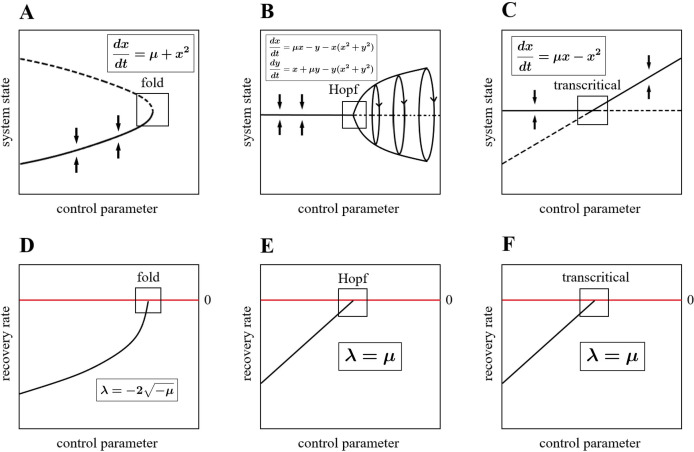
(A–C) The normal forms of fold, (supercritical) Hopf, and transcritical bifurcation where bifurcation occurs at μ=0 (hollow square). Their mathematical expressions are inside the boxes within the corresponding figures. (D–F) The recovery rate λ of the normal forms for the fold, (supercritical) Hopf and transcritical bifurcation as a function of the control parameter μ, their function expressions are inside the boxes within the corresponding figures. Bifurcation occurs when the recovery rate λ reaches zero (hollow square).

The bifurcation theory is a classical and widely used mathematical tool to understand tipping points. According to the centre manifold theorem [7], as a high-dimensional dynamical system approaches a bifurcation, its dynamics converges to a lower-dimensional space which exhibits dynamics topologically equivalent to those of the normal form of that bifurcation [7]. Here, we focused on codimension-one bifurcations in continuous-time dynamical systems, including the fold, Hopf and transcritical bifurcation. Many tipping points of complex systems from nature and society are initiated by these three types of bifurcation [18,40]. The normal forms of fold, Hopf and transcritical bifurcation are shown in figure 2A–C, respectively.

Suppose dx/dt=f(x,μ(t)) has quasi-static attractor $x^{*}(\mu )$ where μ is the bifurcation parameter. Then the recovery rate is defined as the maximal real part of the eigenvalues of the Jacobian matrix when $x=x^{*}(\mu )$ in an n-dimensional dynamical system [41,42]


$$\begin{array}{lcr} & \begin{array}{r} \lambda =\max\{Re\{eigvals\left({\left[\begin{array}{cccc} \frac{\partial f_{1}}{\partial x_{1}} & \frac{\partial f_{1}}{\partial x_{2}} & \cdots & \frac{\partial f_{1}}{\partial x_{n}}\\ \frac{\partial f_{2}}{\partial x_{1}} & \frac{\partial f_{2}}{\partial x_{2}} & \cdots & \frac{\partial f_{2}}{\partial x_{n}}\\ \vdots \; & \vdots \; & \ddots & \vdots \;\\ \frac{\partial f_{n}}{\partial x_{1}} & \frac{\partial f_{n}}{\partial x_{2}} & \cdots & \frac{\partial f_{n}}{\partial x_{n}} \end{array}\right]}_{x=x^{*}(\mu )}\right)\}\}, \end{array} & \end{array}$$

where eigvals refers to the operation of computing eigenvalue and Re refers to the operation of taking the real part. Note that when the recovery rate of a system shifts from negative value to positive value, the bifurcation occurs [7]. In other words, if we know the relation between the recovery rate of a system and the bifurcation parameter, we can easily identify the tipping point for this system. It is also worth noting that the relation between the recovery rate and the bifurcation parameter is the same for the systems of the same bifurcation type in normal forms, as shown in figure 2D–F, respectively. We assume that our DL algorithm can detect features of the recovery rate for the system by training it on a training set generated from a sufficiently diverse library of possible dynamical systems with fold, Hopf and transcritical bifurcation. Then the DL algorithm can be used to predict the tipping points where the recovery rate becomes zero based on the detected relation between the recovery rate and the bifurcation parameter of the system. Note that we only focus on dynamical systems exhibiting fold, Hopf and transcritical bifurcation in this article. In order to predict tipping points of other bifurcation types, we can expand the training library by including simulated data exhibiting those dynamics.

### 2.2. Generation of training data for the DL algorithm

We construct two-dimensional dynamical systems of the following form which are used to generate the training data based on simulation [18]


(2.1) $$\begin{array}{r} \frac{dx}{dt}=\sum_{i=1}^{10}a_{i}p_{i}(x,y)\\ \frac{dy}{dt}=\sum_{i=1}^{10}b_{i}p_{i}(x,y), \end{array}$$

where x and y are state variables, p(x,y) is a vector containing all polynomials in x and y from zero up to third order


$$p(x,y)=(1,x,y,x^{2},xy,y^{2},x^{3},x^{2}y,xy^{2},y^{3}),$$

$p_{i}(x,y)$ is the i-th component of p(x,y). $a_{i}$ and $b_{i}$ are parameters randomly drawn from standard normal distribution, and then half of these parameters are selected at random and set to zero. The parameters for the cubic terms are set to the negative of their absolute values to encourage models with bounded solutions. Since our training data is required to contain representation of all possible dynamics that may occur in the study time series data, we generate models with different sets of parameter values in equation (2.1) until a required number of each type of bifurcation (fold, Hopf or transcritical) has been discovered.

After a model is generated, we perform numerical simulation of this model with 10 000 time steps from a randomly drawn initial condition and test whether the system converges to an equilibrium point. The odeint function from the Python package Scipy [43] is applied in the numerical simulation with a step size of 0.01. The criterion that we used to determine the convergence is that the maximum difference between the final 10 points in numerical simulation is less than $10^{-8}$. The convergence is required in order to search for bifurcations that occur at non-hyperbolic equilibria [7]. The models that do not converge are discarded. For the models that converge, we apply AUTO-07P [44] to identify bifurcations along the equilibrium branch by either increasing or decreasing each nonzero parameter. Note that AUTO-07P is applied to noise-free models. For each bifurcation identified, we first set the initial condition with the value of the equilibrium of the model and a burn-in period of 100 units of time. Additive white or red noise is introduced into the model equations. Then we run simulations of the model with noise and obtain the quasi-static attractor time series with noise and bifurcation parameter time series which are used for training. We also run simulations of the same model without noise and obtain the corresponding quasi-static attractor time series and bifurcation parameter time series which are used for calculating the recovery rate λ to locate the bifurcation point where λ changes from negative value to positive value,


(2.2) $$\begin{array}{r} \lambda =\max\{Re\{eigvals\left({\left[\begin{array}{cc} \frac{\partial (\sum_{i=1}^{10}a_{i}p_{i}(x,y))}{\partial x} & \frac{\partial (\sum_{i=1}^{10}a_{i}p_{i}(x,y))}{\partial y}\\ \frac{\partial (\sum_{i=1}^{10}b_{i}p_{i}(x,y))}{\partial x} & \frac{\partial (\sum_{i=1}^{10}b_{i}p_{i}(x,y))}{\partial y} \end{array}\right]}_{\left(x,y)=(x^{*},y^{*}\right)}\right)\}\}. \end{array}$$

The simulations of the models with noise are based on the Euler Maruyama method [45] with a step size of 0.01 and the simulations of the models without noise are based on the Euler method [46] with the same step size.

For each model where a bifurcation is identified, we perform five independent simulations of the model with varying bifurcation parameters from its initial value to a terminal value beyond the bifurcation point given by AUTO-07P. In these five simulations, we set the changing rate of the bifurcation parameter to remain constant within each simulation where the changing rate of the bifurcation parameter is the change in the bifurcation parameter per unit time with a step size of 0.01 but vary across different simulations. The ratio of each changing rate of the bifurcation parameter to the smallest changing rate of the bifurcation parameter in these five simulations is drawn from 1,2,…,10. The nonzero changing rate of the bifurcation parameter can lead to the delay in the occurrence of tipping points, which is called rate-delayed tipping [20,21]. Due to rate-delayed tipping, the theoretical bifurcation point given by AUTO-07P is not the accurate location of the tipping point. Therefore, we identify the location of the tipping point by the recovery rate equation (2.2) and use this identified tipping point as the training label for our supervised DL training process (electronic supplementary material, note S2). If no tipping point is detected in a simulation, this simulated data will be discarded. Otherwise, we randomly and irregularly sample $l_{s}$ points from quasi-static attractor time series with noise which is simulated by the variable x of the model and corresponding bifurcation parameter time series between the initial value and the tipping point, where $l_{s}$ is drawn from Uniform(505,1000). Then we select the first 500 points as training data. The obtained training data can make the DL algorithm learn the features of recovery rate from time series data with different changing rates of parameter and varying distances between the end of the time series and the tipping points. The training data generation process is illustrated in electronic supplementary material, figure S19.

Here, we train the DL algorithm on datasets with white and red noise. The white noise in simulation has the amplitude drawn from a triangular distribution centred at 0.01 with upper and lower bounds 0.0125 and 0.0075, respectively, while the red noise is modelled by the AR [1] process in which the lag-1 autoregressive coefficient is between −1 and 1. It is worth noting that the simulations shift to a new regime before the bifurcation point due to the noise [47,48]. Since the location of this premature transition is stochastic, we still use the location where the quasi-static attractor without noise loses stability as the training label for the DL algorithm even if it is not accurate. We mathematically illustrate how slight perturbation causes bifurcation to occur earlier and its stochasticity in electronic supplementary material, note S6.

### 2.3. DL algorithm architecture and training

In this article, we use the two-dimensional CNN-LSTM DL algorithm [49,50]. The two-dimensional CNN-LSTM architecture is shown in figure 1. We use a train/validation/test split of 0.95/0.04/0.01 for the DL algorithm, and Mean Squared Error (MSE) as the loss function to minimize the difference between the real tipping points (training labels) and the predictions. The DL algorithm is trained on 3 00 000 instances for 200 epoches. We train ten neural networks and report the performance of the DL algorithm averaged over them. The hyperparameters of the DL model are tuned through random search, and we present the optimal hyperparameters of the DL model in electronic supplementary material, table S1.

Before training our DL algorithm, there are several preprocessing matters [51]. The quasi-static attractor time series with noise are detrended using Lowess smoothing with a span of 0.2 to obtain the residual time series used for training [52]. Besides, in order to obtain the robustness of the DL algorithm on time series of various lengths, we zero out the left side of the residual time series and corresponding bifurcation parameter time series, with the length drawn from Uniform(0,250). Due to the zeroing process, the DL algorithm can deal with time series even if it is a little short. After the zeroing process, each residual time series is normalized by dividing each time series data point by the average absolute value of the residuals across the non-zero part of the time series. In addition, each bifurcation parameter time series is also normalized: each data point in the time series minus the initial value of the bifurcation parameter time series, and then divided by the distance between the initial and final values of the bifurcation parameter time series, thereby mapping the bifurcation parameter time series to the interval of [0,1]. The corresponding label, i.e. the tipping point, is normalized following the same procedure as the normalization of the data point in bifurcation parameter time series. Thus the relative distance of the tipping point to the time series in the training set for the DL algorithm (calculated as the tipping points minus the final value of the bifurcation parameter time series, divided by the distance between the initial and final values of the bifurcation parameter time series) is between 0.01 and 2. In other words, the labels in the training set, after normalization, will fall within the interval of [1.01,3].

### 2.4. Theoretical models used for testing

The simulation of theoretical models with noise is based on the Euler Maruyama method [45] with a step size of 0.01 (δt=0.01 units of time) unless otherwise stated. The amplitude of white noise σ is 0.01 while the red noise is modeled by the AR [1] process in which the lag−1 autoregressive coefficient ϕ is drawn from Uniform(−1,1). We set five different changing rates of the bifurcation parameter in simulations of each theoretical model. The bifurcation point of each simulation is the location where the recovery rate changes from negative value to positive value. Then we sample from these simulations to obtain regularly sampled model time series and irregularly sampled model time series for testing. The lengths of these model time series range from 250 to 500, and the relative distances of tipping points to these time series range from 0.01 to 2.

We apply the relative error ε of tipping points prediction to evaluate the performance of the DL algorithm, which is defined as [25]


$$\begin{array}{lcr} & \varepsilon =\frac{\left|{\hat{\mu }}_{c}-\mu_{c}\right|}{\left|\mu_{\text{end}}-\mu_{c}\right|}, & \end{array}$$

where ${\hat{\mu }}_{c}$ is the predicted tipping point, $\mu_{c}$ is the real tipping point, and $\mu_{\text{end}}$ is the value of the bifurcation parameter at the last point of the test time series, representing the parameter value in the series that is closest to the bifurcation point. The relative error is designed to compare prediction errors across different systems whose bifurcation parameters differ in orders of magnitude.

#### 2.4.1. Theoretical models with white noise

To test the fold bifurcation with white noise, we use the May’s harvesting model [29]. This is given by


$$\begin{array}{lcr} & \frac{dx}{dt}=rx(1-\frac{x}{k})-\frac{hx^{2}}{s^{2}+x^{2}}+\sigma \xi (t), & \end{array}$$

where x represents biomass of some population, k is its carrying capacity, h is the harvesting rate, s characterizes the nonlinear relationship between harvesting output and current biomass, r is the intrinsic per capita growth rate of the population, and ξ(t) is a Gaussian white noise process. We use parameter values r=1, k=1, s=0.1 and h increases at rates of $1\times 10^{-5},2\times 10^{-5},\ldots ,5\times 10^{-5}$. We generate model time series by this equation from 11 initial values of h, which are 0,0.02,0.04,…,0.18,0.2. In this configuration, the fold bifurcation occurs in h∈[0.2713,0.2926].

To test the Hopf bifurcation with white noise, we use a three-species chaotic food chain model [30]. This is given by


$$\begin{array}{lcr} & \begin{array}{rl} & \frac{dr}{dt}=r(1-\frac{r}{k})-\frac{x_{c}y_{c}cr}{r+r_{0}}+\sigma \xi_{r}(t),\\ & \frac{dc}{dt}=x_{c}c(\frac{y_{c}r}{r+r_{0}}-1)-\frac{x_{p}y_{p}pc}{c+c_{0}}+\sigma \xi_{c}(t),\\ & \frac{dp}{dt}=x_{p}p(\frac{y_{p}c}{c+c_{0}}-1)+\sigma \xi_{p}(t), \end{array} & \end{array}$$

where r, c and p represent the population densities of the resource, consumer and predator species, respectively. k characterizes the environmental capacity of the resource species. $x_{c}$, $y_{c}$, $x_{p}$, $y_{p}$, $r_{0}$ and $c_{0}$ are other parameters in the system. $\xi_{r}(t)$, $\xi_{c}(t)$ and $\xi_{p}(t)$ are independent Gaussian white noise processes. For our simulations, we use parameter values $x_{c}=0.4$, $y_{c}=2.009$, $x_{p}=0.08$, $y_{p}=2.876$, $r_{0}=0.16129$, $c_{0}=0.5$ and k increases at rates of $1\times 10^{-5},2\times 10^{-5},\ldots ,5\times 10^{-5}$. We generate model time series by these equations from 11 initial values of k, which are 0.20,0.22,0.24,…,0.38,0.40. In this configuration, the Hopf bifurcation occurs in k∈[0.4656,0.4839].

To test the transcritical bifurcation with white noise, we use the Rozenzweig–MacArthur consumer-resource model [31]. This is given by


$$\begin{array}{lcr} & \begin{array}{rl} & \frac{dx}{dt}=gx(1-\frac{x}{k})-\frac{axy}{1+ahx}+\sigma \xi_{x}(t),\\ & \frac{dy}{dt}=\frac{eaxy}{1+ahx}-my+\sigma \xi_{y}(t), \end{array} & \end{array}$$

where x and y represent the population densities of the resource, consumer, respectively. g is the intrinsic per capita growth rate of x, k is its carrying capacity, a is the attack rate of y, e is the conversion factor, h is the handling time, m is the per capita consumer mortality rate, and $\xi_{x}(t)$ and $\xi_{y}(t)$ are independent Gaussian white noise processes. We fix the parameter values r=4, k=1.7, e=0.5, h=0.15, m=2 and a increases at rates of $1\times 10^{-4},2\times 10^{-4},\ldots ,5\times 10^{-4}$. We generate model time series by these equations from 11 initial values of a, which are 0,0.5,1.0,…,4.5,5.0. In this configuration, the transcritical bifurcation is a∈[5.8819,5.8823].

#### 2.4.2. Theoretical models with red noise

To test the fold bifurcation with red noise, we use a climate model describing the temperature of an ocean on a spherical planet subjected to radiative heating [32,33] which can simulate a transition from a greenhouse to an icehouse Earth [53]. The model is given by


$$\begin{array}{lcr} & \begin{array}{rl} & \frac{dT}{dt}=\frac{-e\rho T^{4}+\frac{1}{4}uI_{0}(1-a_{p})}{c}+\eta (t),\\ & \text{with}\;a_{p}=a-bT, \end{array} & \end{array}$$

where T represents the average temperature of ocean, u is relative intensity of solar radiation, e is effective emissivity, $I_{0}$ is solar irradiance, c is a constant thermal inertia, and $a_{p}$ is the planetary albedo. a and b are parameters that define a linear feedback between ice and albedo variability and temperature. η(t) is a red noise process with lag−1 autoregressive coefficient ϕ. We use e=0.69, $I_{0}=71944000$, $c=10^{8}$, a=2.8, b=0.009, ρ=0.03, δt=1 time unit and u decreases at rates of $5\times 10^{-7},6\times 10^{-7},\ldots ,9\times 10^{-7}$. We generate model time series by this equation from 11 initial values of u, which are 1.4,1.38,1.36,…,1.22,1.2. In this configuration, the fold bifurcation occurs in u∈[0.9596,0.9631].

To test the Hopf bifurcation with red noise, we use the middle Pleistocene transition system [34], explaining the dynamics of glacial cycles, which is given by


$$\begin{array}{lcr} & \begin{array}{rl} & \frac{dx}{dt}=-x-y+\eta_{x}(t),\\ & \frac{dy}{dt}=-pz+uy+sz^{2}-yz^{2}+\eta_{y}(t),\\ & \frac{dz}{dt}=-q(x+z)+\eta_{z}(t), \end{array} & \end{array}$$

where x, y and z represent the global ice volume, the atmospheric greenhouse gas concentration and the deep ocean temperature, respectively. All variables are rescaled to dimensionless form, p=1, q=1.2, s=0.8 are parameters, and u increases at rates of $1\times 10^{-5},2\times 10^{-5},\ldots ,5\times 10^{-5}$. $\eta_{x}(t)$*,* $\eta_{y}(t)$ and $\eta_{z}(t)$ are independent red noise processes with lag−1 autoregressive coefficient ϕ. We generate model time series by these equations from 11 initial values of u, which are 0,0.03,0.06,…,0.27,0.3. In this configuration, the Hopf bifurcation occurs in u∈[0.3546,0.3547].

To test the transcritical bifurcation with red noise, we use the simplified version of the TRIFFID dynamic global vegetation model [35,54]. It can be used to simulate the dieback of the Amazon rainforest [54], which is mainly driven by climate change [55]. The model is given by


$$\begin{array}{lcr} & \begin{array}{rl} & \frac{dV}{dt}=PV^{*}(1-V)-GV+\eta (t), \end{array} & \end{array}$$

where V represents the broadleaf fraction, G is a disturbance coefficient (0.004 /year) and $V^{*}$ is either the value of V or 0.1 if V falls below 0.1. η(t) is a red noise process with lag−1 autoregressive coefficient ϕ. P is the productivity, in dimensionless area fraction units, it decreases at rates of $1\times 10^{-5},2\times 10^{-5},\ldots ,5\times 10^{-5}$. We generate model time series by this equation from 11 initial values of P, which are 0.90,0.82,0.74,…,0.18,0.10. In this configuration, the transcritical bifurcation occurs in P∈[−0.0061,−0.0046].

#### 2.4.3. Theoretical models with hysteresis phenomenon

We also test our DL model on irregularly sampled model time series generated by two theoretical neuroscience models with white noise. These systems exhibit hysteresis, which suggests that when the bifurcation parameter changes in opposite directions, these systems will undergo sudden shifts at different bifurcation points. The ascending arousal system is modelled in terms of the neuronal populations and their interactions [36], which is given by


$$\begin{array}{lcr} & \begin{array}{rl} & \tau_{v}\frac{dV_{v}}{dt}=-V_{v}+v_{vm}Q_{m}+D+\sigma \xi_{v}(t),\\ & \tau_{m}\frac{dV_{m}}{dt}=-V_{m}+v_{ma}Q_{a}+v_{mv}Q_{v}+\sigma \xi_{m}(t). \end{array} & \end{array}$$

Each population j=v,a,m*,* where v is ventrolateral preoptic area, a is acetylcholine, and m is monoamines. Each population j has a mean cell body potential $V_{j}$ relative to resting and a mean firing rate $Q_{j}$. The relation of $Q_{j}$ to $V_{j}$ is described by $Q_{j}=Q_{max}/[1+exp(-(V_{j}-\theta )/\sigma )]$, where $Q_{max}$ is the maximum possible rate, equal to 100$s^{-1}$. Besides this, $V_{a}$ is constant, $\tau_{j}$ is the decay time for the neuromodulator expressed by group j, the $v_{jk}$ weights the input from population k to j, $\xi_{v}(t)$ and $\xi_{m}(t)$ are independent Gaussian white noise processes. The model parameters are consistent with physiological and behavioural measures, θ=10mV, σ=3mV, $v_{ma}Q_{a}=1$mV, $v_{vm}=v_{mv}=-1.9$mVs and $\tau_{m}=\tau_{v}=10$s. In our simulations within the interval of [0.1,1.9]*,* we force the parameter D to increase from 0.1 or decrease from 1.9 at a rate of 1/7200mV per unit time. In this configuration, a fold/fold-hysteresis loop occurs at D=1.153 (in the increasing direction) and D=0.883 (in the decreasing direction), respectively.

Another system is the Sprott B system [56] with a single excitation, which can be used for researching bursting oscillation in neuroscience [37]. The model is given by


$$\begin{array}{lcr} & \begin{array}{rl} & \frac{dx}{dt}=a(y-x)+\sigma \xi_{x}(t),\\ & \frac{dy}{dt}=xz+\beta \;\cos(k)+\sigma \xi_{y}(t),\\ & \frac{dz}{dt}=b-xy+\sigma \xi_{z}(t), \end{array} & \end{array}$$

where a=8, b=2.89 and β=5. $\xi_{x}(t)$, $\xi_{y}(t)$ and $\xi_{z}(t)$ are independent Gaussian white noise processes. We force the parameter k to increase from π or decrease from 2π at a rate of $\pi \times 10^{-3}$ within the interval of [π,2π]. A Hopf/Hopf-hysteresis bursting occurs at k=1.461π (in the increasing direction) and k=1.539π (in the decreasing direction), respectively, in this configuration.

### 2.5. Empirical systems used for testing

In this work, we use three empirical examples in the fields of microbiology, thermoacoustics, and climatology to evaluate the performance of the DL algorithm.

In the first example, photo-inhibition drives cyanobacterial population to a fold bifurcation when a critical light level is exceeded [38]. In this system, bifurcation parameter light irradiance starts at 477 μmol photons $m^{-2}s^{-1}$ and is increased in steps of 23 μmol photons $m^{-2}s^{-1}$ each day. The time series includes 7784 data points spanning overall 28.86 days with time interval equal to 0.0035 day (5 min).

The second example is a thermoacoustic system where positive feedback between the unsteady heat release rate fluctuations and the acoustic field in the confinement can result in a transition to high amplitude limit cycle oscillations [57]. Induja *et al.* [20] conduct experiments in a thermoacoustic system exhibiting Hopf bifurcation. They pass several constant mass flow rates of air through a horizontal Rijke tube which consists of an electrically heated wire mesh in a rectangular duct and increase the voltage applied across the wire mesh to attain the transition to thermoacoustic instability. In such cases, they observe that the occurrence of Hopf bifurcation depends on the changing rate of control parameter. We select three experimental sets for our study with the bifurcation parameter of voltage increasing at rates of 20, 40 and 60 mV/s. The voltage ranges from 0 to 2.4V. The corresponding time series data consists of 1 200 000, 600 000 and 4 00 000 data points, respectively.

The third example is an Atlantic Meridional Overturning Circulation (AMOC) tipping event simulated by the Community Earth System Model (CESM; version 1.0.5) [39]. The data represent a time series of the AMOC strength at 26°N and a depth of 1000 m driven by a slowly varying freshwater flux forcing in the North Atlantic over the region between latitudes 20 and 50°N. The freshwater flux forcing is increased with a rate of $3\times 10^{-4}$ Sv year${,}^{-1}$ until model year 2200.

### 2.6. Comparison of DL algorithm with competing algorithms

For detecting EWS in quasi-static attractor time series with white noise, H. Held and T. Kleinen [9] develop a technique called degenerate fingerprinting using PCA to approximate the critical mode, then estimating the lag−1 autoregressive coefficient of the critical mode as an indicator (electronic supplementary material, note S5). Boettner and Boers [10,58] propose an unbiased estimate of the lag−1 autoregressive coefficient for time series with red noise which we refer to as the BB method (electronic supplementary material, note S5). Florian Grziwotz *et al.* [12] introduce an EWS robust to limited level of noise, named DEV, which is rooted in bifurcation theory of dynamical systems to estimate the dominant eigenvalue of the system (electronic supplementary material, note S5). After obtaining EWS through these three approaches, linear regression or nonlinear fit between EWS and bifurcation parameters is used to make an extrapolation to anticipate the occurrence of tipping points. In our comparative experiment, we employ linear regression or fit a quadratic curve for extrapolation, and then we select the estimate with the better performance.

It is worth noting that the required information of detecting EWS with degenerate fingerprinting is different from that with our DL algorithm. The degenerate fingerprinting requires information from all variables of the study system to approximate the critical mode, whereas our DL algorithm only requires data from one variable of the study system. Similarly, the DEV also requires data from one variable of the study system. Moreover, the BB method is designed for estimating the lag-1 autoregressive coefficient ofone-dimensional systems, whereas our DL algorithm is suitable for analysing multidimensional systems. The DEV can also deal with multidimensional systems.

## 3. Results

### 3.1. Performance of DL algorithm on model time series

**Figure 3.**
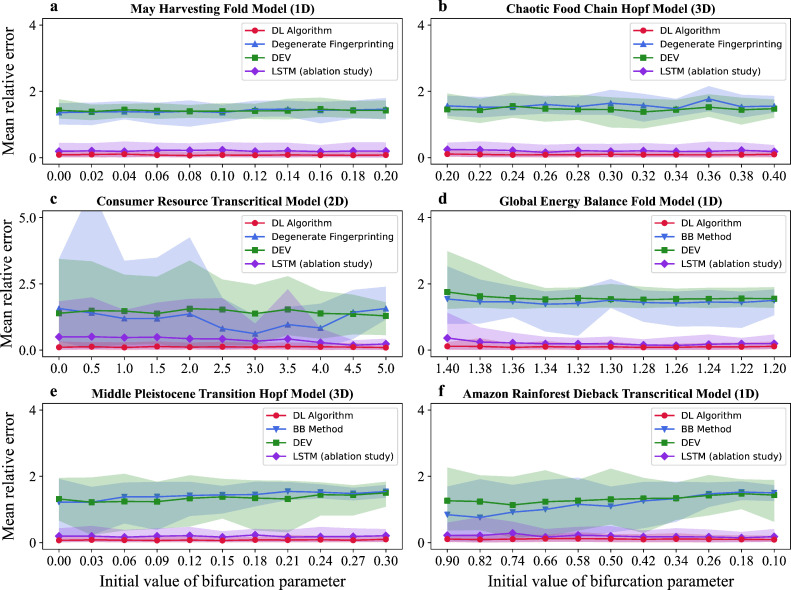
We tested the DL model on regularly sampled model time series with 11 initial values of bifurcation parameter. Here the mean relative error of tipping points prediction is plotted as line graphs against the initial value of the parameter. The shaded area represents the 90% percentile interval (from the 5th to 95th percentile) for the relative error in predicting tipping points. (a–c) We compared the DL algorithm (red lines) with degenerate fingerprinting (blue lines), DEV (green lines) and LSTM (purple lines) on three ecological model time series with white noise. These model time series undergo fold, Hopf and transcritical bifurcation, respectively. (d–f) The DL algorithm (red lines) is compared with the BB method (blue lines), DEV (green lines) and LSTM (purple lines) on three climate model time series with red noise. These model time series undergo fold, Hopf and transcritical bifurcation, respectively.

We applied the DL model on regularly sampled time series generated from three ecological models with white noise and three climate models with red noise. We generated test data for each model by changing the bifurcation parameter with 11 different initial values and five different changing rates. We generated 10 test time series for each initial value and changing rate of the bifurcation parameter (50 test time series for each initial value). To evaluate the performance of the DL algorithm, we measured the relative error of tipping points prediction for each of the 50 test time series associated with every initial value of the bifurcation parameter. The performance of the DL algorithm is evaluated by the mean relative error of tipping points prediction, which is averaged over the 50 measurements of the relative error of tipping points prediction for each initial value of the bifurcation parameter. We designed an ablation study by dropping out the two-dimensional CNN layer, with LSTM serving as a competing algorithm, using the same two-feature input (residual and bifurcation parameter) for a fair comparison. We first compared the results of the DL algorithm with those of degenerate fingerprinting [9], DEV [12] and LSTM in three ecological models with white noise which exhibit fold, Hopf and transcritical bifurcations, respectively. Then we compared the results of the DL algorithm with those of the BB method [10], DEV [12] and LSTM in three climate models with red noise which exhibit fold, Hopf and transcritical bifurcations, respectively. As figure 3 shows, the DL algorithm outperforms the other competing algorithms for all initial values of each model. Moreover, the DL algorithm exhibits smaller fluctuations of relative error of tipping points prediction than the other competing algorithms for each initial value and exhibits smaller fluctuations of the mean relative error of tipping points prediction than the other competing algorithms across different initial values. Our results suggest that the DL algorithm is more robust against different initial values of the bifurcation parameter than competing algorithms. The mean relative errors of our DL algorithm and the four competing algorithms on regularly sampled model time series are presented in electronic supplementary material, table S4.

**Figure 4.**
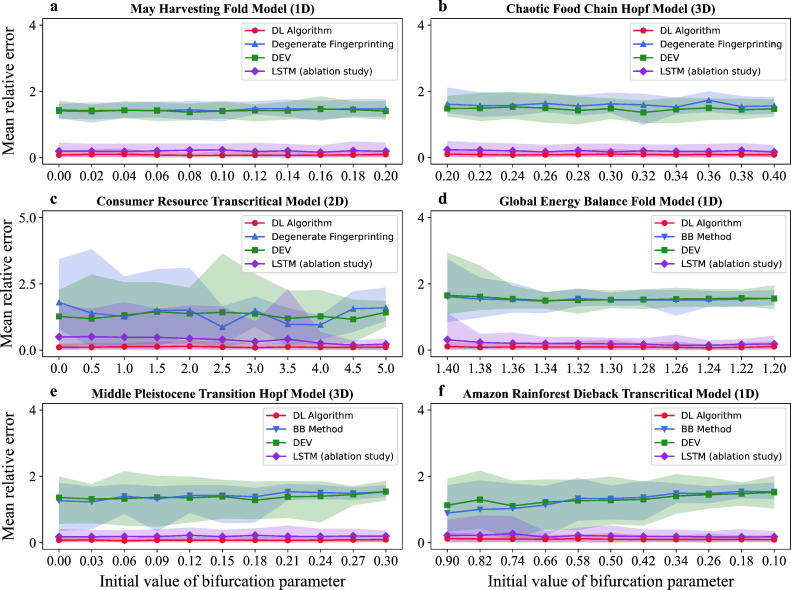
We tested the DL model on irregularly sampled model time series with 11 initial values of bifurcation parameter. Here, the mean relative error of tipping points prediction is plotted as line graphs against the initial value of the parameter. The shaded area represents the 90% percentile interval (from the 5th to 95th percentile) for the relative error in predicting tipping points. (a–c) We compared the DL algorithm (red lines) with degenerate fingerprinting (blue lines), DEV (green lines) and LSTM (purple lines) on three ecological model time series with white noise. (d–f) The DL algorithm (red lines) is compared with the BB method (blue lines), DEV (green lines) and LSTM (purple lines) on three climate model time series with red noise. We used linear interpolation to transform these irregularly sampled time series into equidistant data so that they are suitable for degenerate fingerprinting, BB method and DEV.

We further tested the DL model on irregularly sampled time series from the above six models. It is worth noting that the above-competing algorithms are not applicable for irregularly sampled time series. Therefore, we used linear interpolation to transform these irregularly sampled time series into equidistant data. This allows us to use the competing algorithms of degenerate fingerprinting, BB method and DEV for detecting EWS on reconstructed model time series [53]. LSTM is also applied as a competing algorithm. From figure 4, we find that the DL algorithm outperforms the other competing algorithms for all initial values of each model. Moreover, our results suggest that the DL algorithm is more robust against different initial values of the bifurcation parameter than competing algorithms. The mean relative errors of our DL algorithm and the four competing algorithms on irregularly sampled model time series are presented in electronic supplementary material, table S5.

In the ablation study, our DL algorithm achieves a mean relative error of about 9% in predicting tipping points on these model time series, while LSTM exhibits a mean relative error of about 23%, as shown in electronic supplementary material, figures S1 and S2. The key difference between our DL algorithm and the LSTM lies in the two-dimensional CNN layer, where the features of the reconstructed state space of the dynamical system are extracted for tipping points prediction. The result that our DL algorithm outperforms LSTM highlights the importance of using features of the reconstructed system for tipping points prediction. We also plotted the line graphs of the mean relative error of tipping points prediction against the distance between the final value of bifurcation parameter time series and the value of the tipping point, as shown in electronic supplementary material, figures S3 and S4. Our results suggest that the DL algorithm is more robust against different distance to the tipping point than competing algorithms. Moreover, we plotted the line graphs of the mean relative error of tipping points prediction against the initial value of the parameter in five different changing rates of the bifurcation parameter, as shown in electronic supplementary material, figures S5 and S6. Our results suggest that the DL algorithm is robust against various changing rates of bifurcation parameters. Furthermore, to evaluate the robustness of our DL algorithm under low-quality data, we conducted a series of experiments with varying degrees of missing values and noise intensity, as shown in electronic supplementary material, figures S11 and S12, respectively. Our results suggest that the DL algorithm maintains stable performance in the presence of limited missing data, especially when the missing rate is as low as 10%, and is also robust to increasing levels of noise intensity.

**Figure 5.**
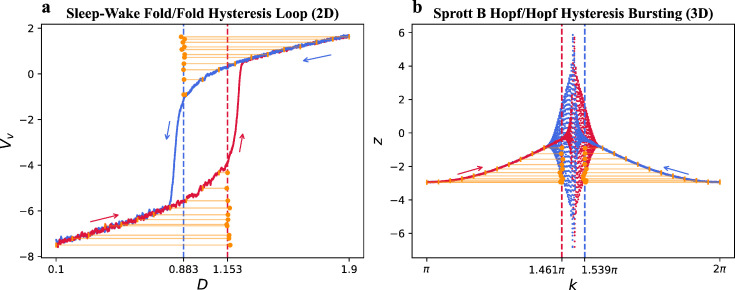
The performance of the DL algorithm in predicting transitions of irregularly sampled time series generated by theoretical models exhibiting hysteresis. The red and blue curves illustrate the time series forced by increasing and decreasing bifurcation parameter, respectively. The orange dots denote the DL predictions, and the orange short vertical lines denote the initial points of the time series data used for prediction. We connect them with orange lines. (a) In the case of the sleep-wake system, a fold/fold hysteresis loop is observed at D=1.153 (red dashed line) and D=0.883 (blue dashed line), respectively. The predicted tipping points are between [1.148,1.169] and [0.8679,0.8898], respectively. (b) For the Sprott B system, a Hopf/Hopf hysteresis bursting occurs at k=1.461π (red dashed line) and k=1.539π (blue dashed line), respectively. The predicted tipping points are between [1.454π,1.464π] and [1.534π,1.545π], respectively.

We then tested the DL model on irregularly sampled time series from the ascending arousal system with a fold/fold hysteresis loop. We conducted irregular sampling of 400 points from 22 initial values of the bifurcation parameter, which are 0.1,0.2,…,1.1 (bifurcation parameter increases from these initial values) and 1.9,1.8,…,0.9 (bifurcation parameter decreases from these initial values). We applied the DL model on these irregularly sampled time series and the mean relative errors of tipping points prediction are 3.12% and 3.31%, respectively, for the increasing and decreasing bifurcation parameter cases, as shown in figure 5a. Similarly, we tested the DL model on irregularly sampled time series from the Sprott B system with a Hopf/Hopf-hysteresis bursting. We irregularly sampled 400 points from 22 initial values of the bifurcation parameter, which are π,1.04π,…,1.4π (bifurcation parameter increases from these initial values) and 2π,1.96π,…,1.6π (bifurcation parameter decreases from these initial values). We applied the DL model on these irregularly sampled time series and the mean relative errors of tipping points prediction are 2.82% and 2.54%, respectively, for the increasing and decreasing bifurcation parameter cases, as shown in figure 5b. Our results suggest that the DL algorithm is effective for tipping point prediction in theoretical models with hysteresis phenomenon.

### 3.2. Performance of DL algorithm on empirical time series

We tested the DL model on three empirical examples, including a cyanobacteria microcosm experiment under light stress [38], a physical experiment of voice oscillation during heat [20], and an AMOC tipping event driven by an increasing freshwater flux forcing [39]. For each empirical system, we applied the DL model to several irregularly sampled time series. Each time series includes 400 data points with different initial values of the control parameter. Then we used linear interpolation to transform these irregularly sampled records to time series with equidistant data. This allows us to use the competing algorithms of degenerate fingerprinting, BB method and DEV for detecting EWS on reconstructed empirical records [53]. Note that the first two empirical examples from ecology and physics are subject to white noise, while the third empirical example from climatology is subject to red noise. Therefore, we applied degenerate fingerprinting to the first two datasets and the BB method to the third. DEV will be applied to all three, as it is applicable to both white and red noise [12].

**Figure 6.**
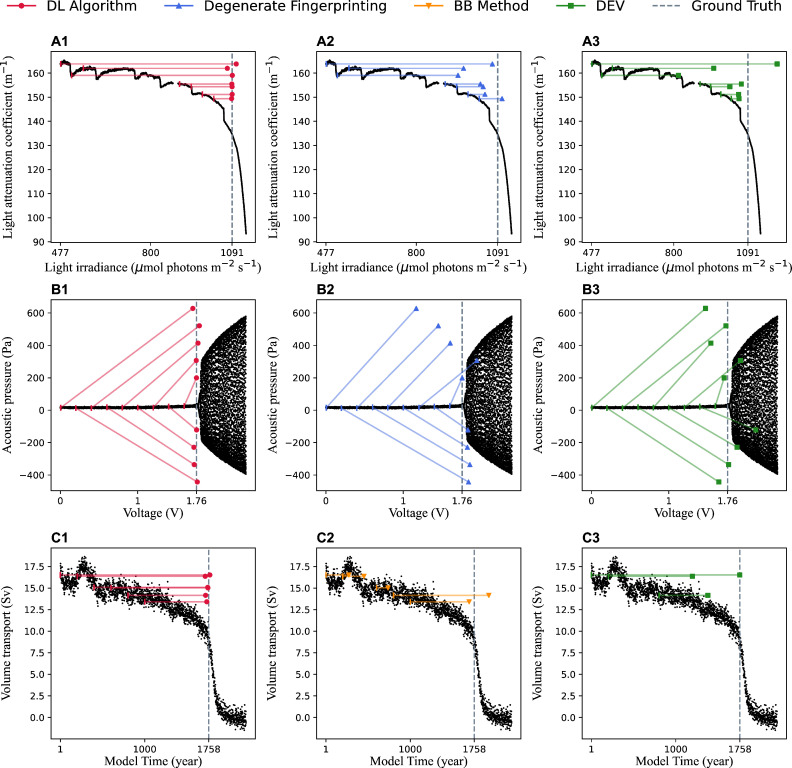
The performance of the DL algorithm in predicting tipping points on irregularly sampled empirical time series. The dots denote the DL predictions, and the short vertical lines denote the initial points of the time series data used for prediction. We connect them with lines. (A1–A3) Cyanobacterial population undergoes a fold bifurcation, which is measured by light attenuation coefficient. The fold bifurcation occurs at 1091 μmol photons $m^{-2}s^{-1}$, while the predictions by our DL algorithm are between 1074 μmol photons $m^{-2}s^{-1}$ and 1106 μmol photons $m^{-2}s^{-1}$. (B1–B3) The thermoacoustic system undergoes a Hopf bifurcation under the 40 mV/s changing rate of the control parameter voltage. The Hopf bifurcation occurs at 1.76 V, while the predictions by our DL algorithm are between 1.71 and 1.79 V. We compared the performance of the DL algorithm (red) with degenerate fingerprinting (blue) and DEV (green). (C1–C3) The AMOC undergoes a fold bifurcation at 1758 year. The predictions by our DL algorithm are between 1717 year and 1771 year. We compared the performance of the DL algorithm (red) with BB method (orange) and DEV (green). We used linear interpolation to transform these irregularly sampled time series into equidistant data so that they are suitable for degenerate fingerprinting, the BB method and DEV.

In the cyanobacteria microcosm experiment under light stress, the photo-inhibition drives a cyanobacterial population, measured by light attenuation coefficient, towards a tipping point with fold bifurcation when a critical threshold of light irradiance is approached [38]. We sampled seven time series with different initial values of light irradiance which are 477, 517, 557, 903, 944, 985 and 1025 μmol photons $m^{-2}s^{-1}$. Since the data between 879 and 903 μmol photons $m^{-2}s^{-1}$ are missing, the time series is sampled with initial values of 477, 517, 557 μmol photons $m^{-2}s^{-1}$, respectively, and the same final value of 879 μmol photons $m^{-2}s^{-1}$. The mean relative error of the prediction with our DL algorithm is 3.82%, where the ground truth of the tipping point is 1091 μmol photons $m^{-2}s^{-1}$. As shown in figure 6A1–A3, the DL algorithm outperforms degenerate fingerprinting and DEV in all cases. These results suggest the robustness of the DL algorithm in tipping points prediction against different initial values of light irradiance.

The second empirical data we analysed is the thermoacoustic system. The state of the thermoacoustic system is measured by acoustic pressure. The thermoacoustic system undergoes a Hopf bifurcation from a non-oscillatory to an oscillatory state with increasing voltage [24,57,59,60]). It has also been demonstrated that the transition to high amplitude limit cycle oscillations occurs later for faster changing rate of voltage, which can be explained by rate-delayed tipping [20,21]. We sampled time series with nine initial values of voltage which are 0,0.2,…,1.6 V under voltage changing rates of 20 and 40 mV/s, and with ten initial values of voltage which are 0,0.2,…,1.8 V under a voltage changing rate of 60 mV/s. We then applied a change-point detection algorithm [61] to detect tipping points of these empirical time series under voltage changing rates of 20, 40 and 60 mV/s, which are 1.72, 1.76 and 1.87 V, respectively. As shown in figure 6B1–B3, we find that the DL algorithm outperforms degenerate fingerprinting and DEV in predicting the tipping points in most cases under the voltage changing rate of 40 mV/s, with mean relative error of 4.31% by our DL algorithm. We further test these algorithms for the other two changing rates of voltage as shown in electronic supplementary material, figure S7, which suggests the DL algorithms also show best performance among all these algorithms. These results suggest that the DL algorithm is robust against various initial values and changing ranges of voltage.

We further analysed an AMOC tipping event. In this case, the freshwater flux anomaly drives the AMOC, measured by AMOC strength, towards a tipping point with fold bifurcation [39]. Since the freshwater flux forcing is increased at a constant rate (see §2), we used age as a substitute for the bifurcation parameter. We sampled six time series with different initial values of age, which are 1, 201, 401, 601, 801 and 1001 year. The mean relative error of the prediction with our DL algorithm is 4.77%, where the ground truth of the tipping point is 1758 year. As shown in figure 6C1–C3, the DL algorithm outperforms the BB method and DEV in most cases. Due to severe deviations from the ground truth under some initial values, the corresponding results of the BB method and DEV are not displayed in the figure. These results suggest the robustness of the DL algorithm in tipping points prediction against different initial values of age.

## 4. Discussion

Predicting the occurrence of tipping points based on time series is a challenging problem. In this work, we develop a DL algorithm that exploits information of normal form from the training systems for tipping points prediction. This algorithm can deal with both regularly sampled and irregularly sampled time series by using the time series sampled from a single variable of a system. Our results show that the DL algorithm not only outperforms traditional methods in the accuracy of tipping points prediction for regularly sampled time series, but also achieves accurate prediction for irregularly sampled time series. Our results pave the way to develop effective strategies to prevent and prepare tipping points from various real-world systems [62].

There are three major advantages of our work. First, traditional methods for tipping points prediction can only deal with regularly sampled time series [9–12]. However, the DL algorithm used in this article can deal with both regularly sampled time series and irregularly sampled time series. This is because the convolution kernels in the two-dimensional CNN layer can extract features of the reconstructed system from segments of irregularly sampled time series (electronic supplementary material, note S3), which is used for tipping points prediction in the LSTM layer. Second, most existing methods [9,25–27,63]) require information on multiple interacting variables in the study system for predicting tipping points. However, based on the embedding theorem for irregular sampling [28], the DL algorithm only requires the time series sampled from a single variable of the study system. Third, the effect of rate-delayed tipping has been ignored in previous methods for tipping points prediction [9–12]. This is due to the limitations of the theory of fast-slow systems used in previous methods. However, we consider the effect of rate-delayed tipping in our work and label the data by using the fact that the quasi-static attractor loses stability at the occurrence of tipping points in our training set (see §2). The DL algorithm trained with such labels is robust against different changing rates of bifurcation parameter (electronic supplementary material, figures S5–S7).

The embedding theorem for irregular sampling [28] requires that the dimension d of the system reconstructed by delay embedding must be larger than twice the dimension m of the study system (electronic supplementary material, note S3). Therefore, the length of convolution kernels d used in the CNN layer of our DL algorithm is required to be larger than 2m. However, due to the time-varying nonstationarity of a dynamical system approaching a bifurcation, the dynamical information of a system can not be precisely captured by a too-long convolution kernel [64]. This prevents us from setting the convolution kernel length d too long, but the condition of the embedding theorem requires d to be larger than twice the system’s dimension m. Therefore, our DL algorithm may not perform well in predicting tipping points in high-dimensional systems. To illustrate this, we set the convolution kernel length d to 30 to satisfy d>2m and retrained the model on the training set to predict the 10-dimensional networked dynamical system. As shown in electronic supplementary material, figure S17, a decrease in prediction accuracy is observed. Another limitation of our DL algorithm is that it requires the existence of tipping points as prior knowledge. If there is no tipping point in the system, the output of the DL algorithm is meaningless. Therefore, several methods such as DEV [12] or some DL classifiers [18,22–24] should be used to determine whether a system is approaching a tipping point first. Then our DL algorithm can be applied to predict the specific location of a tipping point if it exists.

To analyse whether the DL algorithm uses the recovery rate in the normal form for prediction, we designed two control experiments. First, we trained three DL models on three datasets, each consisting solely of time series with fold, Hopf, and transcritical bifurcation, respectively. Then we applied these three DL models on irregularly sampled model time series from six theoretical models. We found that the DL model trained on a dataset containing the features of a bifurcation performed better in predicting tipping points of test time series with that bifurcation, compared with another two trained on datasets without the corresponding normal form features (electronic supplementary material, figure S8). These results suggest that the DL algorithm can extract the features of normal form. Second, we trained two DL models on two datasets, each consisting solely of time series with supercritical and subcritical pitchfork bifurcation, respectively (electronic supplementary material, note S7). Then we applied these two DL models on irregularly sampled model time series from a model with supercritical pitchfork, where both models achieved similar performance in predicting the tipping points (electronic supplementary material, figure S9). The normal forms of supercritical and subcritical pitchfork bifurcations exhibit the same relation between the recovery rate and the bifurcation parameter, and only differ in the cubic term. Thus, the DL model trained on time series with subcritical pitchfork bifurcation can be used to predict tipping points of time series with supercritical pitchfork bifurcation, which suggest that the DL algorithm can extract the features of the recovery rate in normal form. In addition to these two control experiments, we computed the saliency scores for the trained DL model when it processes irregularly sampled model time series. As shown in electronic supplementary material, figure S13, for each theoretical model, the curves of saliency scores consistently exhibit maximum peaks near the beginning and the end of the time series, indicating that the DL model assigns greater importance to the initial and final segments of the input time series when making predictions. To be more specific, the DL algorithm extracts the features of recovery rate near the beginning and the end of the time series, along with their temporal dependencies, to locate the tipping points where the recovery rate approaches zero.

Beyond the prediction of codimension-one bifurcations, the approach of training DL models on a training set generated from a sufficiently diverse library of possible dynamical systems to predict tipping points in previously unseen systems has the potential to be extended to other local bifurcations, such as Bogdanov–Takens bifurcation [7]. We presented an illustrative example by predicting the Bogdanov–Takens bifurcation in the Hindmarsh–Rose neuron model [65] (see electronic supplementary material, note S9 and figure S16). However, whether the DL model trained in this manner can predict a broader range of codimension-two bifurcations remains to be further explored. Furthermore, the approach is not limited to the specific DL architecture employed in this study. Transformer-based models like Informer are highly suitable for predicting tipping points due to their ability to model long-range dependencies, accelerate parallel computation, and robustly handle irregularly sampled data. We further validated it by training the Informer model [66] on the same training set (see electronic supplementary material, note S10). We then evaluated its performance on six theoretical systems (electronic supplementary material, figure S18). The results demonstrate that our method exhibits good applicability across different DL architectures, providing valuable insights for future research.

Our work raises several problems worthy of future pursuit. First, we have studied the situation of local codimension-one bifurcations in continuous-time dynamical systems in this paper. For future work, we can focus on tipping points prediction in discrete-time dynamical systems, such as period-doubling bifurcation [7] which arise naturally in physiology [67,68] and ecology [69]. It would be interesting to develop a method that can deal with both regularly sampled and irregularly sampled time series for systems with period-doubling bifurcation. Second, it would be interesting to investigate the prediction of tipping points when the system undergoes a change in global stability, leading it from monostability to multistability [70]. Third, one can develop interpretable machine learning models for tipping points prediction by combining dynamical system theory with neural networks [71–75], which offer an avenue for making safe and reliable high-stakes decisions for policy makers [76].

## Data Availability

All the simulated datasets and the empirical datasets used to test the deep learning algorithm have been deposited in Github [77] and the training set used to train the deep learning algorithm has been deposited in Zenodo [78]. Supplementary material is available online [79].
